# pH-sensitive CAP/SiO_2_ composite for efficient co-delivery of doxorubicin and siRNA to overcome multiple drug resistance†

**DOI:** 10.1039/c9ra07894k

**Published:** 2020-01-27

**Authors:** Zheng Cai, Yuezhu Chen, Yingwen Zhang, Zhimei He, Xiaoge Wu, Li-Ping Jiang

**Affiliations:** State Key Laboratory of Analytical Chemistry for Life Science, School of Chemistry and Chemical Engineering, Nanjing University Nanjing 210023 P. R. China jianglp@nju.edu.cn; School of Pharmacy, Nanjing Medical University Nanjing 211166 P. R. China

## Abstract

Long-term administration of chemotherapeutic agents often leads to multiple drug resistance (MDR), which greatly impairs the treatment outcome. To overcome this problem, a biodegradable nanocarrier based on an acid-sensitive calcium phosphate/silica dioxide (CAP/SiO_2_) composite was constructed for the codelivery of drug and siRNA. Anticancer drug doxorubicin (DOX) was encapsulated into the composite scaffold by interacting with the exposed Ca^2+^ of CAP/SiO_2_ to achieve high drug loading (180 μg mg^−1^). With further decoration of siRNA, the nanocarrier was applied to enhance the therapeutic efficacy by silencing MDR-relevant genes (P-gp) of DOX-resistance K562/ADR cancer cells. Benefiting from the intrinsic acid degradability of CAP/SiO_2_, the nanocomposite demonstrated pH-responsive release behavior, favoring drug/siRNA release within acidic endo-/lysosomes. Consequently, due to the drug and gene effects, this biodegradable nanomedicine demonstrated enhanced therapeutic efficiency, providing a novel strategy for cancer therapy.

## Abstract 2

CAP/SiO_2_ composite with good biocompatibility and acid biodegradability has been prepared. The proposed drug and gene codelivery system based on it demonstrated enhanced therapeutic efficacy for multiple drug resistance cells.

## Graphical abstract

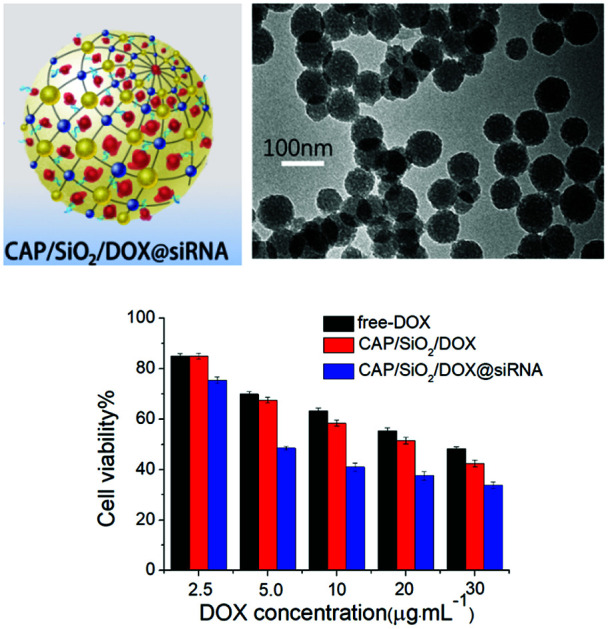

## Introduction

Cancer has become a major threat to human beings due to its high incidence, severe mortality, and low survival rate. Besides traditional surgical operation, chemotherapy plays an important role as a primary and supportive care for cancer treatment. However, current chemotherapy still faces severe challenges including side effects and drug-resistance. Multiple drug resistance (MDR) is one of the major limitations in cancer therapy. MDR decreases the accumulation of drug within the cell and increases the repairing mechanism of DNA damage leading to low therapeutic efficiency of single agents. The efflux pump mediated drug resistance is predominantly attributed to the over-expression of P-glycoprotein (P-gp) and multidrug resistance-associated protein (MRP) which may pump the anticancer drugs out of the cancer cells.^1,2^ RNA interference with small interfering RNA (siRNA) that mediates gene-silencing phenomena has shown great potential as tactics for a number of diseases. Contemporaneously, delivering siRNA and drug could achieve a combination effect of chemotherapy and gene therapy. Nevertheless, the clinical use of siRNA is hindered by three major problems: (1) rapid enzymatic degradation-induced short blood circulation; (2) poor cellular uptake; (3) insufficient tissue bioavailability.^3–6^ It is difficult for naked siRNA to reach tumour sites and successfully enter cells, which means that it needs a carrier system to exert the biological activity of siRNA, especially, in *in vivo* application. Therefore, to stabilize the siRNA in systemic circulation and to ensure its maximum accumulation in cancer tissue, an effective drug delivery carrier is urgently desired.

To date, various codelivery platforms for siRNA and chemotherapy agent have been established to resolve the problem of MDR in cancer cells.^7–10^ As a naturally nontoxic biomineral, calcium phosphate (CAP) featuring acid-degradation, biocompatibility, and simple chemical composition, shows great potential for biomedical applications, such as serving as a vehicle for gene delivery.^11^ CAP formed complexes with the nucleic acid backbone and imparted a stabilizing function to certain DNA structures. Then the complexes passed across the cell membrane *via* ion channel mediated endocytosis.^12^ Usually, the CAP/DNA complex was prepared *via* co-precipitation. However, the rapid growth of calcium phosphate crystal was hard to control, and resulted in the formation of large agglomerates, which drastically reduced the complex quality and transfection efficiency, inspiring us to develop new strategy to prepare CAP-based composite materials.^13–15^

**Scheme 1:**
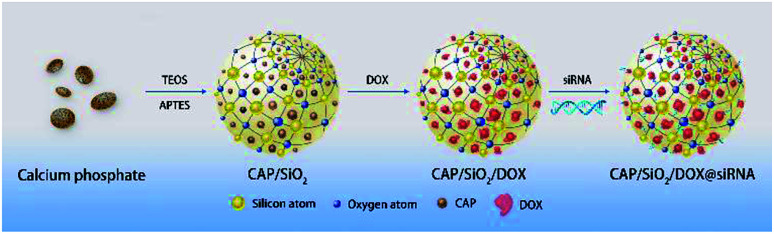
Illustration of the construction of the drug delivery system.

In this paper, a novel drug delivery system (DDS) with good biocompatibility and pH sensitivity was developed as depicted in Scheme 1. Colloid CAP was embedded into the net structure of SiO_2_, forming CAP/SiO_2_ composite through reverse microemulsion method. Anticancer drug doxorubicin (DOX) was then encapsulated into the CAP/SiO_2_ composite in the form of Ca^2+^–DOX complex.^16^ Furthermore, P-pg-targeted small interfering RNA (siRNA) was absorbed into the CAP/SiO_2_ composite *via* electrostatic interaction. The prepared CAP/SiO_2_ composite could protect siRNA due to the high affinity of Ca^2+^ ion to the phosphate groups in the nucleic acids.^17^ The prepared nanocarrier exhibited an excellent pH-responsive release property, for the CAP component in CAP/SiO_2_ was easily dissolved in lower pH environment. As a result, drug and siRNA could be released from the carrier in a weak acid condition. This codelivery system has been tested in human chronic myelogenous leukaemia K562/ADR cell line, which is drug-resistance cell strain. Cell experimental results suggested that DOX and siRNA codelivery by this nanocarrier could have synergistic effect in treatment in K562/ADR cell line. A greater *in vitro* cytotoxic effect was observed with the siRNA/DOX codelivery compared to free-DOX in K562/ADR cell line. This proposed codelivery system might be a promising approach in MDR tumour cell lines in the future.

## Results and discussion

### Fabrication and characterization the CAP/SiO_2_ composite

**Fig. 1:**
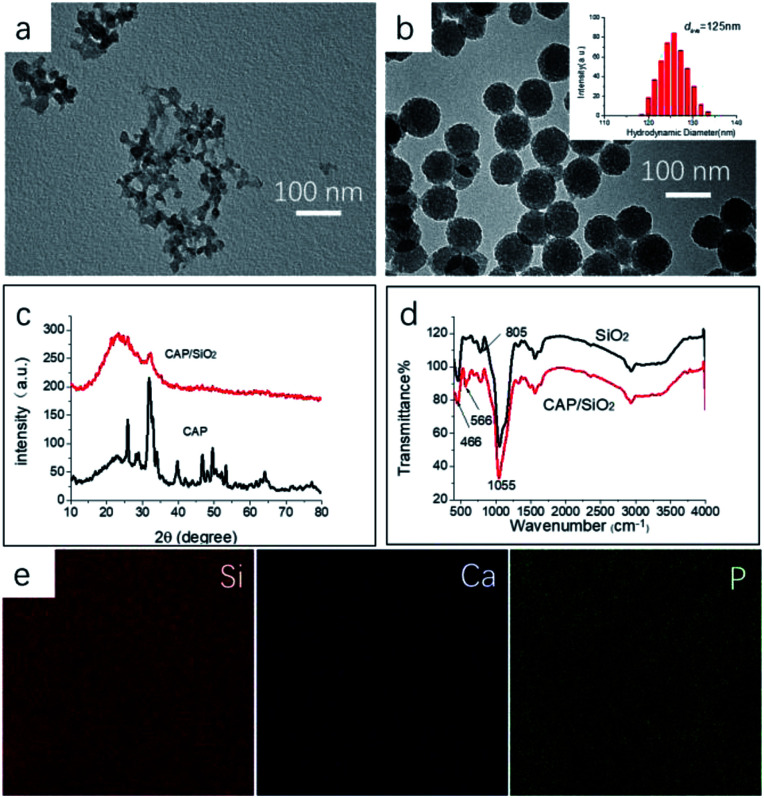
Characterization of CAP and CAP/SiO_2_ composite. The TEM image of obtained CAP (a) and CAP/SiO_2_ composite (b) inset: dynamic light scattering diameter of CAP/SiO_2_ composite. (c) XRD patterns of CAP and CAP/SiO_2_ (d) FT-IR spectra of SiO_2_, CAP/SiO_2_. The element distribution of the surface of CAP/SiO_2_ by EDX (e).

CAP/SiO_2_ composite were prepared using a reverse microemulsion method and further characterized by TEM, XRD and IR. The CAP colloid was prepared with one-step method in water–ethanol liquid, as shown in Fig. 1a, generating CAP/SiO_2_ composite with coarse surface and uniform size around 100 nm in Fig. 1b. Dynamic light scattering (DLS) was used to determine the size distribution of particles. The particles have narrow size distribution with mean size of 125 nm, and the poly dispersity index (PDI) less than 0.2, indicating that the particles were homogeneous. To investigate the chemical composition, XRD patterns of pure CAP and CAP/SiO_2_ composite at room temperature were recorded in Fig. 1c. The generated sharp peaks ranging from 15 degree to 40 degree were attributed to the hexagonal hydroxyapatite (JCPD 09-0432). CAP/SiO_2_ showed only one hump peak and a small peak located at about 31 degree, indicating that CAP has been incorporated into the skeleton of SiO_2_ during the synthesis process.^13,18^ On the other hand, in the FT-IR spectra in Fig. 1d, absorption peaks in the framework region at about 1050, 805, and 466 cm^−1^ were observed, which were attributed to the stretching vibrations of Si–O–Si, bending vibrations of O–Si–O, and rocking vibrations of Si–O–Si, respectively,^18^ further confirming the successful preparation of the CAP/SiO_2_ composite. To analyse the elemental component of CAP/SiO_2_, SEM elemental-mapping was conducted. As shown in Fig. 1e, Si, Ca and P signals were detected to be distributed in the particles, indicating that the CAP precursors were uniformly embedded into the silica network. The O, Si, Ca and P signals were also investigated by XPS (Table 1), and the atomic ratio of Ca and Si was determined to be about 0.45 : 1 (mole ratio).

**Table 1:** Element containing of CAP/SiO_2_ by XPS

Name	Si 2p	Ca 2p	P 2p	O 1s	N 1s
Atom	25.78	11.80	8.79	52.78	1.39

The fabrication mechanism of the CAP/SiO_2_ composite was also investigated. In this work, a reverse microemulsion (water/oil) method was adopted to embed CAP into SiO_2_ skeleton to form CAP/SiO_2_ nanohybrids. Cyclohexane served as a continuous phase in which tetraethyl orthosilicate (TEOS) was dissolved. Microscale water pools containing CAP colloid were stabilized by Triton X-100 and hexyl alcohol. Under the catalysis of ammonia, the TEOS underwent hydrolysis at the oil/water interface, forming Si–O–Si–O continuous network structure to encapsulate CAP.^19^ It was found that the amount of CAP colloid was crucial to the preparation of homogenous CAP/SiO_2_ composite. Pure silicon sphere-like CAP/SiO_2_ composite with smooth surface were obtained (Fig. S1a†) when a small amount of CAP was added. As the amount of CAP increased, the surface became coarse (Fig. S1b†). However, if excessive CAP was added, separated CAP and silica would be observed as shown in Fig. S1c and d.† The reason might be that: as the amount of negatively charged CAP colloid in water phase increased, more CAP were pushed out of the silica intermediate due to the electrostatic repulsion. The surface charge is another important factor that influences the heterogeneous nucleation of CAP/SiO_2_ composite. Here, positively charged aminopropyltriethoxysilane (APTES) was used to reduce the negatively charge density of the silica intermediates and promote the stable incorporation of more CAP into the composite.^20^ Furthermore, the amino group in APTES molecule also catalysed the hydrolysis reaction, accelerated their reaction process and rapidly increased the viscosity of the SiO_2_ spheres, which also helped to trap the CAP colloid within the microspheres. As shown in Fig. S2,† monodispersed CAP/SiO_2_ composite particles were prepared when the volume ratio TEOS to APTES was 1 : 1 (Fig. S2a†). However, excessive APTES increased the viscosity and caused agglomeration (Fig. S2b and c†).

### Drug loading and pH controlling release study

CAP/SiO_2_ composite was proved to be an ideal nanocarrier for drug delivery that CAP on the surface provided a large number of anchoring points large for drug molecules, wherein Ca^2+^ ion was chelated with DOX to form Ca^2+^–DOX complex.^16^ Ca^2+^ ion had a high affinity with DOX than ordinary static electricity, which avoided non-specific adsorption and early drug release. The supernatant of CAP/SiO_2_ was detected by UV-vis (Fig. S3†), and the decreasing absorbance indicated the successful formation of the CAP/SiO_2_/DOX composite. The proposed CAP/SiO_2_ composite had good loading capability that encapsulation of DOX was up to 90% and the amount of DOX loaded into the CAP/SiO_2_ was determined to be 180 mg g^−1^.

**Fig. 2:**
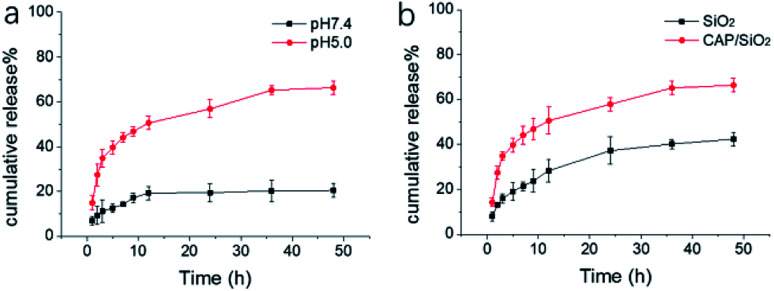
Mass percentage of cumulative released DOX from (a) CAP/SiO_2_ in buffer solution (pH 7.4 and 5.0) (b) CAP/SiO_2_ and SiO_2_ in pH 5.0 buffer solution.

The pH-responsive degradability of the CAP/SiO_2_ composite was demonstrated by the following experiments. Firstly, the release rate of DOX from CAP/SiO_2_ composite in different pH solutions (pH 7.4 and 5.0) were measured to mimic the microenvironment of healthy tissue and cellular lysosomes, respectively. The released DOX was quantified by UV-vis absorbance at 480 nm within 48 h. As shown in Fig. 2a, the release amount reached to 67% after DOX-loaded CAP/SiO_2_ dispersed in pH 5.0 for 48 h compared to that of 20% in pH 7.4 buffer solution, indicating good stability in a physiological environment. Upon exposure to pH 5.0 buffer, the cumulative release of DOX from CAP/SiO_2_ composite was much faster than that in pure SiO_2_ (Fig. 2b), indicating that the degradation rate of pure silica with stable network structure was much slower than that of CAP/SiO_2_ composite.^21^ The reason was that, the embedded CAP in the CAP/SiO_2_ composite was dissolved to release Ca^2+^ ion under weak acidic condition. With the Ca^2+^ ion entering into solution, the DOX was also removed from the original composite, which not only facilitated the DOX release, but also promoted the degradation of the composite.^22^ The degradation process of CAP/SiO_2_ in acid buffer solution (pH 5.0) was also monitored by TEM (Fig. S4†). At first stage, the nanoparticles decomposed at the periphery with small pieces of debris shed from the surface. As time went on, the particle size became smaller and smaller until all composites were broken down into pieces, demonstrating that the prepared CAP/SiO_2_ had fast pH responsive behaviour and good biodegradability.

### Delivery of CAP/SiO_2_/DOX@siRNA complex into cells

**Fig. 3:**
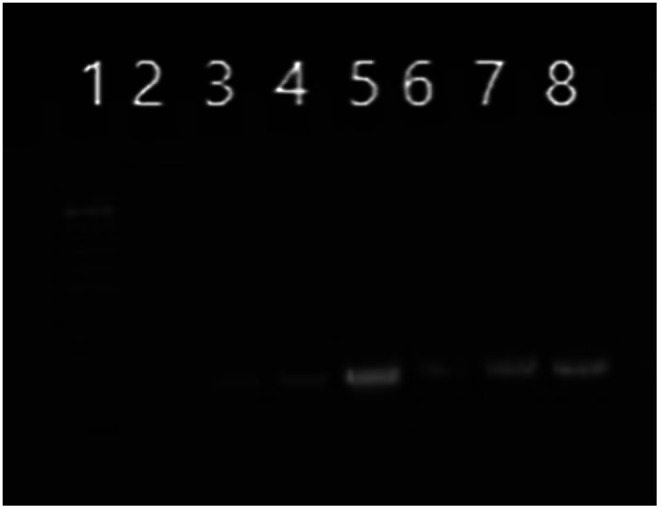
Agarose gel electrophoresis of CAP/SiO_2_/DOX to which siRNA was complexed at various composite to siRNA ratios. Lane 1 was for molecular weight mark, lane 5 was for siRNA only, lane 2, 3, 4, 6, 7, 8 was for the supernatant at different ratios of composite to siRNA, 25 : 1, 20 : 1, 16 : 1, 10 : 1, 8 : 1, 4 : 1, respectively.

CAP/SiO_2_/DOX@siRNA complex was then prepared by adsorbing siRNA on the surface of CAP/SiO_2_/DOX composite *via* electrostatic interaction (Fig. S5†). The strong interaction between calcium ions and negatively-charged phosphate enabled the formed complex to construct a stable inner core for siRNA encapsulation.^12^ As shown in Fig. 3, when the mass ratio of the composite to siRNA increased to 20 : 1, almost all of the siRNA was absorbed on the complex surface. When the ratio of composite to siRNA was further increased to 25 : 1, the line of siRNA disappeared, indicating that there was no residual siRNA in supernatant.

**Fig. 4:**
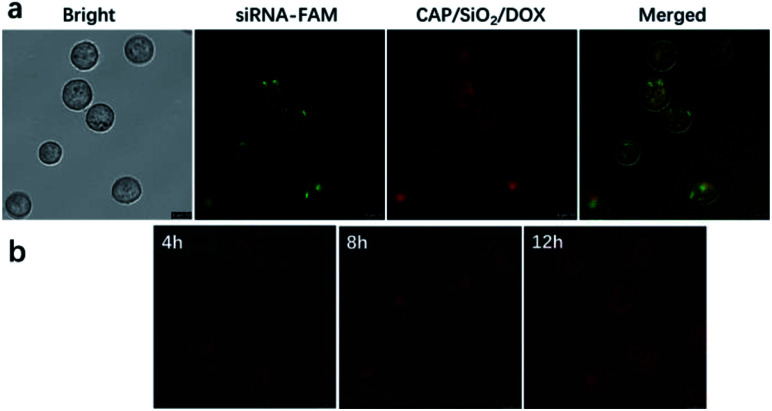
Confocal microscopy images of K562/ADR cells (a) following treatment of CAP/SiO_2_/DOX@siRNA-FAM for 12 h; (b) following treatment of CAP/SiO_2_/DOX@siRNA for 4 h, 8 h, and 12 h, respectively. The concentration of DOX was 20 μg mL^−1^.

Next, we investigated the intracellular delivery of siRNA and DOX using our nano-complex. K562 and K562/ADR cells lines were selected as DOX-sensitive and DOX-resistant cell lines, respectively. The IC_50_ values of free DOX towards K562 and K562/ADR were 0.5 and 40 μg mL^−1^, respectively. We incubated the K562/ADR cells with DOX-loaded CAP/SiO_2_@siRNA-FAM (containing DOX 20 μg mL^−1^) nanocarriers for 12 h. As shown in Fig. 4a, the green fluorescence from siRNA-FAM and the red fluorescence from DOX were all observed in the cytoplasm of K562/ADR cells, indicating that our nano-complex have delivered both the drug and siRNA into the cells.^9^ As shown in Fig. 4b, CAP/SiO_2_/DOX@siRNA were incubated with K562/ADR cells for 4 h, 8 h, and 12 h, the red fluorescence intensity increased along with the incubation time, indicating gradual internalization of CAP/SiO_2_/DOX@siRNA, subsequent disintegration and the following DOX release. By co-staining with DAPI molecules, it was found that CAP/SiO_2_/DOX@siRNA was mostly distributed in the cytoplasm (Fig. S6†).

### Therapeutic effects of the drug delivery system in living cells

**Fig. 5:**
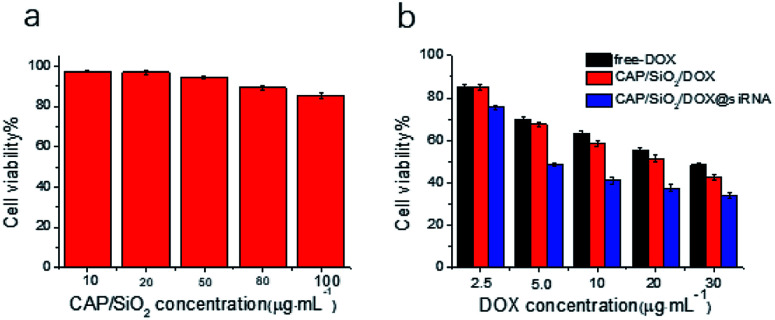
Biocompatibility of nanocarriers towards A562/ADR cells, the percentage of viable cells was estimated using MTT cell proliferation assay. *In vitro* cytotoxicity effect of (a) blank CAP/SiO_2_ composite; (b) free DOX, CAP/SiO_2_/DOX and CAP/SiO_2_/DOX@siRNA against K562/ADR cell of incubation.

The therapeutic efficacy of the prepared nanocarriers was estimated using 3-(4,5-dimethylthiazol-2-yl)-2,5-diphenyltetrazolium bromide (MTT) assay. As shown in Fig. 5a, no significant cytotoxicity of CAP/SiO_2_ composite was observed even at a high concentration of 100 μg mL^−1^, indicating good biocompatibility of CAP/SiO_2_ composite. K562/ADR cells were further treated with free DOX, CAP/SiO_2_/DOX and CAP/SiO_2_/DOX@siRNA for 24 h. All the materials contained equivalent DOX concentration. The cell viability decreased as the concentration of DOX increased, indicating a dosage-dependent cytotoxicity (Fig. 5b). The CAP/SiO_2_/DOX@siRNA containing 30 μg mL^−1^ DOX, causing 77% cell death, which might be ascribed to the fact that the siRNA knocked down the expression of P-gp protein and sensitized the drug toxicity and therefor favoured the therapy.

### Cells apoptosis assay

**Fig. 6:**
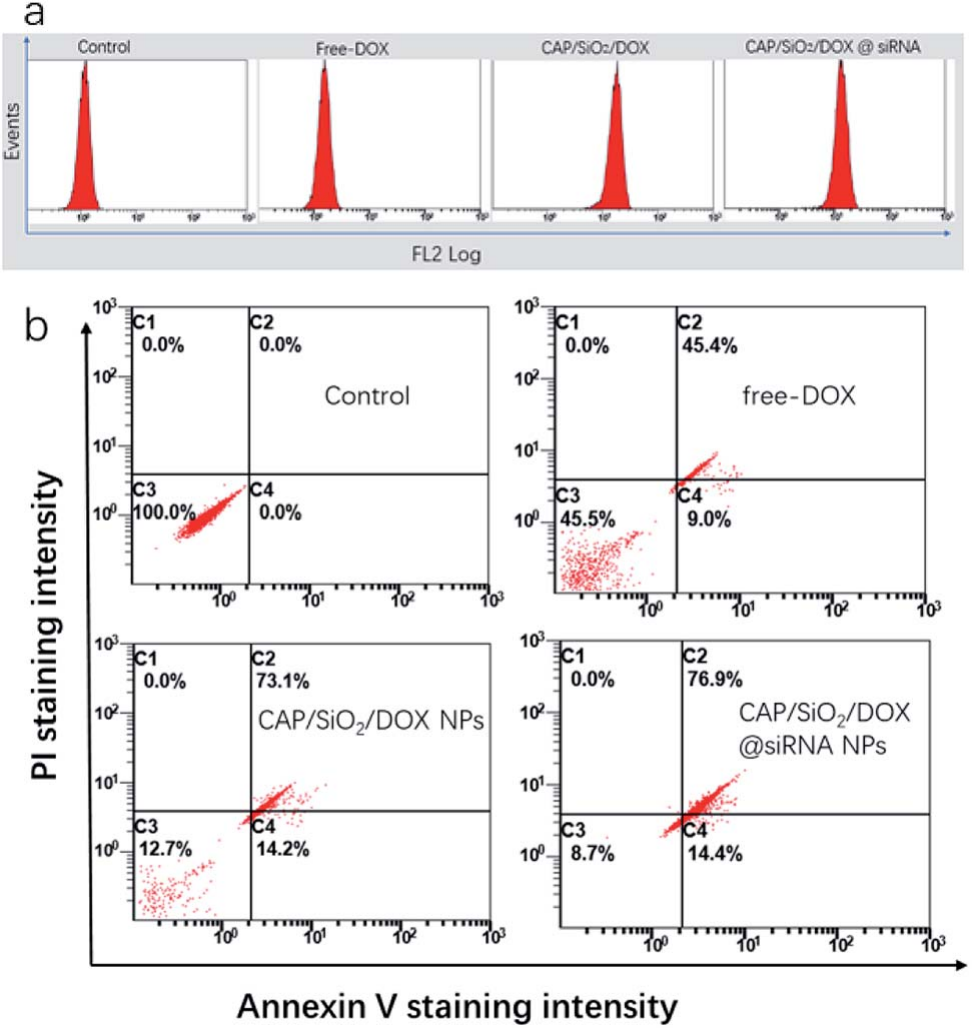
Flow cytometry analysis (a) intracellular fluorescence intensity of DOX; (b) cell apoptosis of K562/ADR after treatments with different formulations at a normalized-DOX concentration (30 μg mL^−1^) for 24 h.

To testify our hypothesis, the synergetic effect of CAP/SiO_2_/DOX@siRNA was investigated using apoptosis kit and western blot. Firstly, the intracellular DOX concentration was determined using flow cytometry analysis (Fig. 6a). The comparatively lower drug uptake after treatment with free DOX was slightly improved by delivering the drug using CAP/SiO_2_ composite. However, in the presence of siRNA, the CAP/SiO_2_ composite delivered more drugs into the cells. It was most likely due to the fact that siRNA down-regulated expression of the MDR1, and may effectively overcome the drug efflux pump mediated drug resistance.

Annexin V-FITC and PI co-staining were performed to assess the survival state of K562/ADR cells more accurately. As shown in Fig. 6b, treatment with free-DOX induced early and late apoptosis of 54.5% towards K562/ADR cells, which further confirmed that K562/ADR cells were resistant to DOX. In comparison, an increase of total apoptosis in K562/ADR cells was observed when treated with CAP/SiO_2_/DOX and CAP/SiO_2_/DOX@siRNA. Many work reported that the liberated Ca^2+^ and PO_4_^3−^ arising from the degradation subsequently destroyed the lysosomal membrane and facilitated the escape of siRNA from lysosomes to cytoplasm to silence gene expression, thus sensitizing the treatment.^12^

### Molecular biology assays

**Fig. 7:**
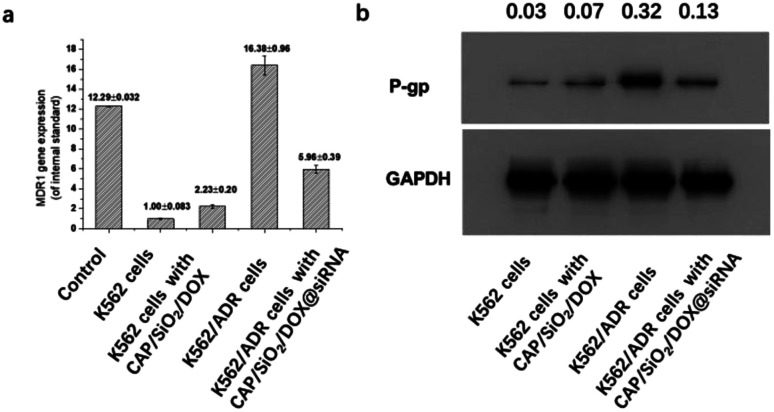
Efficacy of CAP/SiO_2_/DOX@siRNA in suppressing MDR1 gene expression in K562 and K562/ADR cells. (a) Suppression on the MDR1 mRNA lever quantified by real-time PCR analysis (*n* = 3); (b) the expression of P-gp protein evaluated by western blot.

The knockdown efficiency was assessed by measuring the levels of siRNA and protein based on the real-time polymerase chain reaction (RT-PCR) and western blot (WB), respectively. As shown in Fig. 7a and b, the levels of MDR1 mRNA and its P-gp protein in K562/ADR cells were higher than those in K562 cells, indicating that they are drug-resistant. After 24 hours of incubation with DOX loaded CAP/SiO_2_ composite, K562 cells showed a slight increase of MDR1 mRNA compared to the untreated K562 cells. In contrast, for K562/ADR cells, the treatment of DOX/siRNA co-loaded CAP/SiO_2_ composite conduced to the significant downregulation of both MDR1 mRNA (Fig. 7a) and its P-gp glycoprotein (Fig. 7b). These results demonstrated that CAP/SiO_2_/DOX@siRNA complexes effectively co-delivered siRNA and DOX into K562/ADR cells, resulting in the down-regulation of P-gp glycoprotein and reversal of overexpression of MDR1.^9,23,24^ The efficient intracellular delivery of CAP/SiO_2_/DOX@siRNA is expected to overcome multiple drug resistance and improve therapeutic efficacy of human chronic leukaemia.

## Conclusions

In this work, we have successfully developed novel pH-sensitive CAP/SiO_2_ composite nanocarrier for drug and gene codelivery. This nanomaterial exhibits narrow size distribution and good stability in physiological environment. In this system, DOX was loaded onto the CAP/SiO_2_ by forming Ca^2+^–DOX complex, while siRNA targeted to P-gp was absorbed on composite *via* electrostatic attraction. This composite material demonstrated high drug loading efficiency and protected the siRNA from being degraded by enzyme in cells. The CAP/SiO_2_/DOX@siRNA delivery system combining chemotherapy and gene therapy exhibited enhanced cellular uptake with higher cytotoxicity towards K562/ADR cells as compared to a single loaded system. Such easy-to-fabricate composite nanomaterial not only featured good biocompatibility and acid biodegradability, but also showed synergistic effect of siRNA and drug in inducing multiple drug resistance cells apoptosis. We believe that the proposed drug/gene codelivery system has potential application in MDR tumour cell lines for clinical trial in future.

## Experimental

### Synthesis CAP/SiO_2_ composite

Calcium phosphate (CAP) was synthesized according to a previous report.^14^ Briefly, 0.1388 g CaCl_2_ was dissolved in 32.5 mL ethanol under magnetic stirring. Then 1.5 mL 0.1 M NaOH aqueous and 3.5 mL deionized water were injected into the ethanol solution. In addition, 12.5 mL of phosphate aqueous solution containing 0.2685 g Na_2_HPO_4_·12H_2_O was poured into above solution. The reaction continued under magnetic stirring for 1 minute. The colloidal product was centrifuged and washed with ethanol and deionized water, then dissolved in water and kept in refrigerator at 4 °C. CAP/SiO_2_ composite was prepared *via* the synchronous modification of functional group in the water-in-oil microemulsion.^19^ In a typical synthesis, 7.5 mL cyclohexane, 1.8 mL hexyl alcohol, 1.77 mL Triton-X and 100 μL water was put together to get reverse microemulsion, then 380 μL calcium phosphate colloidal solution was put into the microemulsion as water phase. After half an hour later, 25 μL TEOS and 25 μL APTES were added, under stirring for another 30 minutes. The 60 μL NH_3_ was added another 40 min later. After stirring for another 22–24 h, the emulsion was broken by acetone. At last, the product was obtained by centrifugation, and washed by ethanol and water twice respectively.

### Drug loading onto the CAP/SiO_2_ composite

Doxorubicin was loaded inside the CAP/SiO_2_ composite as follows: 1.0 mg CAP/SiO_2_ composite was added to the solution of DOX solution (200 μg mL^−1^, 1 mL), for 24 h while gently shaking at speed of 180 rpm at room temperature, followed by centrifugation to remove any free DOX. The supernatant was detected by UV-vis spectra to determine the quantity of DOX left in solution.

The encapsulation efficiency of DOX (EE) was calculated as the equation:
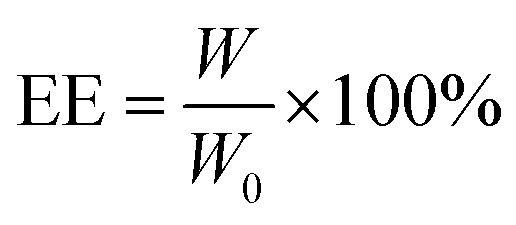
where *W* was the weight of DOX in the particle after centrifugation, *W*_0_ was the weight of DOX initially added in particle preparation.

The drug-loading capacity (DL) was calculated using the following equation
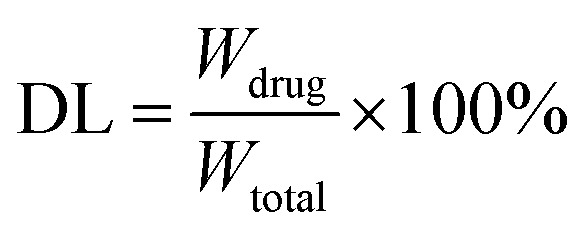
where *W*_drug_ was the weight of DOX and *W*_total_ was the weight of drug and particle together.

### Drug release experiments

To investigate the effect of DOX release at different pH values, the loading drug particles were dispersed in 1 mL aqueous buffer solution (10 mM acetate buffer, pH = 5.0 and 10 mM PBS pH = 7.4) at room temperature. Supernatant were taken from the suspension at predetermined time intervals and other fresh 1 mL buffer solution was replenished. The concentration of released DOX was determined by measuring with UV-vis spectra.

### Fabrication of CAP/SiO_2_/DOX@siRNA codelivery system

The siRNA was captured by composite *via* electrostatic attraction. The complex CAP/SiO_2_/DOX@siRNA was prepared by mixing the siRNA with different the amounts of composite for 60 min at room temperature. The residual siRNA in supernatant was monitored by agarose gel electrophoresis.^25^ Briefly, 10 μL siRNA solution (20 μM) was mixed with 10–65 μg CAP/SiO_2_/DOX in DEPC water. The mass ratios of composite to siRNA were from 4 : 1, 8 : 1, 10 : 1, 16 : 1, 20 : 1 and 25 : 1.

### Cell experiments

#### Confocal laser scanning microscopy imaging

With DOX self-fluorescence, CAP/SiO_2_/DOX@siRNA in K562/ADR cells was further investigated by confocal laser scanning microscopy (CLSM Lecia TCS SP5 confocal microscope, Germany). In brief, K562/ADR cells were seeded on confocal dishes at a density of 1.2 × 10^4^ cells per well. After overnight incubation, the medium was replaced by fresh medium, containing of CAP/SiO_2_/DOX@siRNA (final DOX concentration was 20 μg mL^−1^). After incubation for 4 h, 8 h and 12 h, the cells were washed with PBS twice to remove the away the unbound composite.

#### Cell viability assay

Cells (1.0 × 10^4^ cells per well) were seeded in 96-well plates and cultured for 12 h. Then free DOX, CAP/SiO_2_/DOX, CAP/SiO_2_/DOX@siRNA were added into the cell culture medium respectively. Following incubation for 48 h, cells were treated with MTT solution (0.5 mg mL^−1^) for 4 h at 37 °C. The supernatants were then removed, and 100 μL DMSO were added to each well. Finally, plates were measured at 570 nm on a microplate reader.

#### Flow cytometry analysis

K562/ADR cells were seeded at 2 × 10^5^ cells per well in 6-well plate and cultured for 24 h. 25 μg mL^−1^ of free DOX, CAP/SiO_2_/DOX and CAP/SiO_2_/DOX@siRNA were added to the cells, respectively. After incubation for 24 h, cells were washed with PBS twice, and stained with fluoresces in Annexin V-FITC/PI were added to the cells, respectively for 15 min in dark. Finally, numbers of apoptotic cells were measured by flow cytometry (Becton, Dickinson and Company, USA).

#### Molecular biology assays

Suppression on the expression of MDR1 gene in transfected K562/ADR cells was evaluated at both mRNA and protein levels. mRNA level gene was detected by real-time PCR assay. Expression of P-gp protein was evaluated by western blot analysis (WB). K562 cells and K562/ADR cells were seeded into cell culture flask overnight, incubated with 20 μg mL^−1^ of CAP/SiO_2_/DOX and CAP/SiO_2_/DOX@siRNA for 24 h, respectively. The details were shown in ESI.†

### Materials

CaCl_2_ (AR), Na_2_HPO_4_·12H_2_O (AR) NaOH (AR) ethanol (AR) were purchased from Nanjing Chemistry Reagent co., Ltd. Tetraethyl orthosilicate (TEOS) and aminopropyltriethoxysilane (APTES) were purchased from Aldrich. Doxorubicin (DOX), 1640 medium, 3-(4,5-dimethylthiazol-2-yl)-2,5-diphenyltetrazolium bromide (MTT) and Annexin V-FITC/PI were purchased from KeyGen Biotech Co. (Nanjing, China). All aqueous solutions were prepared using DEPC treated ultrapure water from a Milli-Q system (Millipore, USA). The siRNA duplex consists of 5′-r(CGGAAGGCCUAAUGCCGAA)dTdT (sense) 5′-r(UUCGGCAUUAGGCCUUCCG)dTdT (antisense) was obtained from Shanghai GenePharma (Shanghai, China). K562 and K562/ADR cells were supplied by KeyGen Biotech Co. Ltd. (Nanjing, China). All the biological regents used in RT-PCR and WB were obtained from KeyGen Biotech Co. Ltd. (Nanjing, China).

## Conflicts of interest

There are no conflicts to declare.

## Supplementary Material

RA-010-C9RA07894K-s001

## References

[cit1] Zheng W. J., Yin T. T., Chen Q. C., Qin X. Y., Huang X. Q., Zhao S., Xu T. Y., Chen L. M., Liu J. (2016). Acta Biomater..

[cit2] Hua J., Mutch D. G., Herzog T. J. (2005). Gynecol. Oncol..

[cit3] Novina C. D., Sharp P. A. (2004). Nature.

[cit4] Elmen J., Thonberg H., Ljungberg K., Frieden M., Westergaard M., Xu Y. H., Wahren B., Liang Z. C., Urum H., Koch T., Wahlestedt C. (2005). Nucleic Acids Res..

[cit5] Chiu Y. L., Ali A., Chu C. Y., Cao H., Rana T. M. (2004). Chem. Biol..

[cit6] Soutschek J., Akinc A., Bramlage B., Charisse K., Constien R., Donoghue M., Elbashir S., Geick A., Hadwiger P., Harborth J., John M., Kesavan V., Lavine G., Pandey R. K., Racie T., Rajeev K. G., Rohl I., Toudjarska I., Wang G., Wuschko S., Bumcrot D., Koteliansky V., Limmer S., Manoharan M., Vornlocher H. P. (2004). Nature.

[cit7] Sokolova V., Epple M. (2008). Angew. Chem., Int. Ed..

[cit8] Saad M., Garbuzenko O. B., Minko T. (2008). Nanomedicine.

[cit9] Li J.-M., Wang Y.-Y., Zhao M.-X., Tan C.-P., Li Y.-Q., Le X.-Y., Ji L.-N., Mao Z.-W. (2012). Biomaterials.

[cit10] Ding J., Liang T., Zhou Y., He Z., Min Q., Jiang L., Zhu J. (2017). Nano Res..

[cit11] Maitra A. (2005). Expert Rev. Mol. Diagn..

[cit12] Qiu C., Wei W., Sun J., Zhang H.-T., Ding J.-S., Wang J.-C., Zhang Q. (2016). Nanoscale.

[cit13] Hu Y.-Y., Yusufoglu Y., Kanapathipillai M., Yang C.-Y., Wu Y., Thiyagarajan P., Deming T., Akinc M., Schmidt-Rohr K., Mallapragada S. (2009). Soft Matter.

[cit14] Tang Q.-L., Zhu Y.-J., Wu J., Chen F., Cao S.-W. (2011). Nanomedicine.

[cit15] Zhang M., Ishii A., Nishiyama N., Matsumoto S., Ishii T., Yamasaki Y., Kataoka K. (2009). Adv. Mater..

[cit16] Zhao Y., Luo Z., Li M., Qu Q., Ma X., Yu S.-H., Zhao Y. (2015). Angew. Chem., Int. Ed..

[cit17] Truong-Le V. L., Walsh S. M., Schweibert E., Mao H.-Q., Guggino W. B., August J. T., Leong K. W. (1999). Arch. Biochem. Biophys..

[cit18] Tang Q.-L., Zhu Y.-J., Duan Y.-R., Wang Q., Wang K.-W., Cao S.-W., Chen F., Wu J. (2010). Dalton Trans..

[cit19] Yang Y. H., Gao M. Y. (2005). Adv. Mater..

[cit20] Hüsing N., Schubert U., Mezei R., Fratzl P., Riegel B., Kiefer W., Kohler D., Mader W. (1999). Chem. Mater..

[cit21] Pohaku Mitchell K. K., Liberman A., Kummel A. C., Trogler W. C. (2012). J. Am. Chem. Soc..

[cit22] Chen X., liu Y., Lin A., Huang N., Long L., Gang Y., Liu J. (2018). Biomater. Sci..

[cit23] Cao N., Cheng D., Zou S., Ai H., Gao J., Shuai X. (2011). Biomaterials.

[cit24] Meng H., Mai W. X., Zhang H., Xue M., Xia T., Lin S., Wang X., Zhao Y., Ji Z., Zink J. I., Nel A. E. (2013). ACS Nano.

[cit25] Ni Q., Zhang F., Zhang Y., Zhu G., Wang Z., Teng Z., Wang C., Yung B. C., Niu G., Lu G., Zhang L., Chen X. (2018). Adv. Mater..

