# Supplementary material for: pH-sensitive CAP/SiO2 composite for efficient co-delivery of doxorubicin and siRNA to overcome multiple drug resistance
Source: RSC Adv. 2020 Jan 27;10(8):4251–7. doi: 10.1039/c9ra07894k (PMC9049068; doi:10.1039/c9ra07894k)
Supplement: RA-010-C9RA07894K-s001 [file RA-010-C9RA07894K-s001.pdf]

Electronic Supplementary Material (ESI) for RSC Advances.
This journal is © The Royal Society of Chemistry 2020

Supporting information

PH-sensitive CAP/SiO_2 Composite for Efficient Co-delivery of Doxorubicin and siRNA to Overcome Multiple Drug Resistance

Zheng Cai,[a,b] Yuezhu Chen,[a] Yingwen Zhang,[a] Zhimei He,[a] Xiaoge Wu,[a] Li-Ping Jiang*[a]

[a] *State Key Laboratory of Analytical Chemistry for Life Science, School of Chemistry and Chemical Engineering, Nanjing University, Nanjing 210023, P.R. China*

E-mail: jianglp@nju.edu.cn

[b] *School of Pharmacy, Nanjing Medical University, Nanjing 211166, P.R. China*

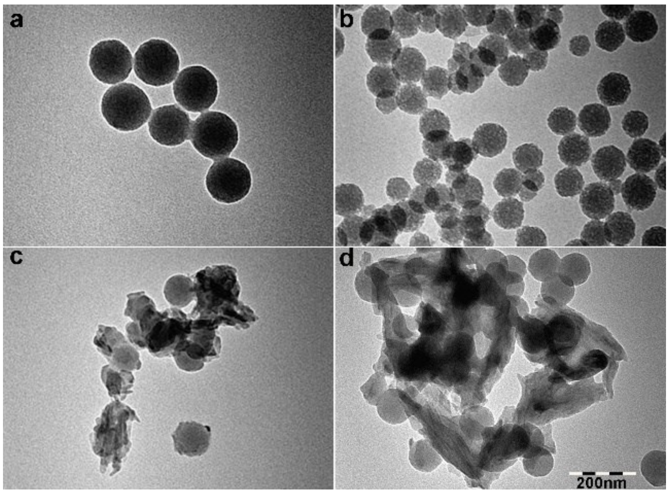

Fig. S1 The TEM images of morphologies of CAP/SiO_2 composite with different ratio of Ca and Si: (a)0.2:1, (b)0.4:1, (c)1:1, (d)2:1 (All the TEM images share the same scale label.)

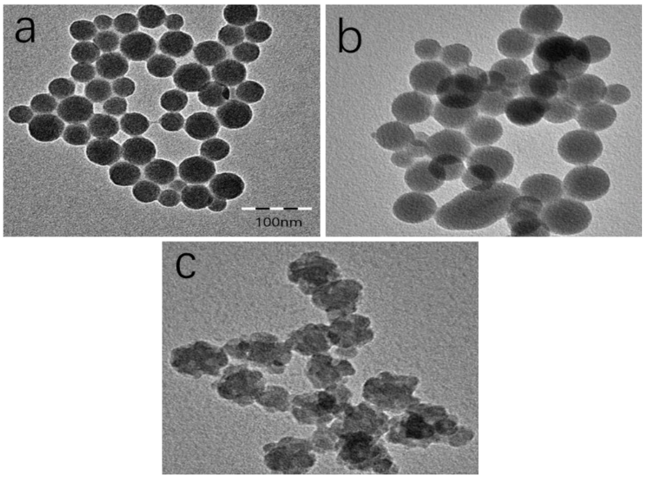

Fig. S2 The TEM images of morphologies of CAP/SiO_2 composite with different ratio of TEOS and APTES. (a) 1:1, (b) 1:1.5, (c)1:2 (*v*:*v*). (All the TEM images share the same scale label.)

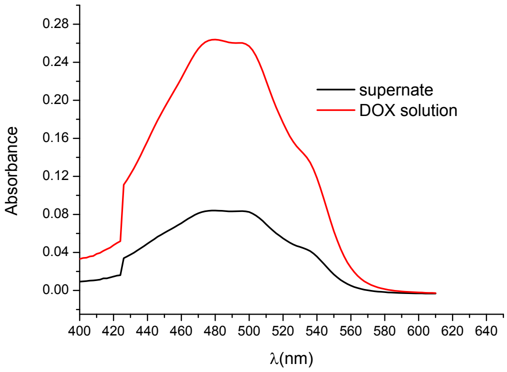

Fig. S3 UV-Vis spectra of DOX solution and the supernatant of CAP/SiO_2 after the loading of DOX.

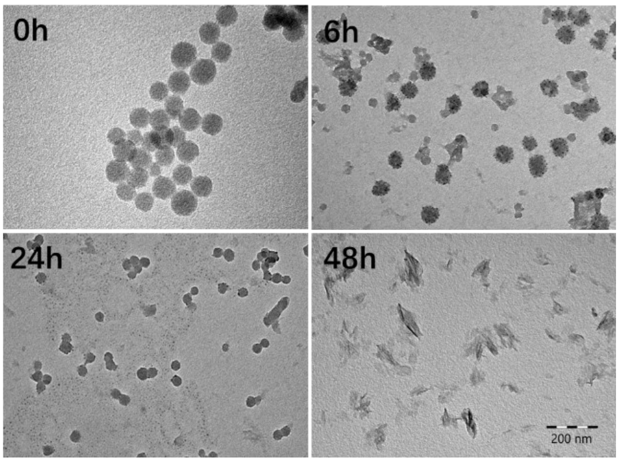

Fig. S4 TEM images of the CAP/SiO_2composite dispersing in acidic buffer solution (pH 5.0) for different times. (All the TEM images share the same scale label)

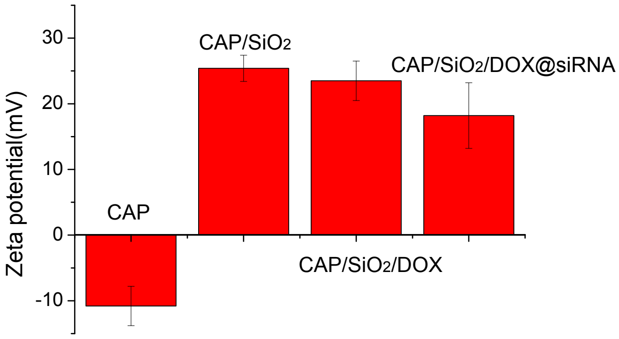

Fig. S5 Zeta potentials of CAP, CAP/SiO_2, CAP/SiO_2/DOX, and CAP/SiO_2/DOX@siRNA.

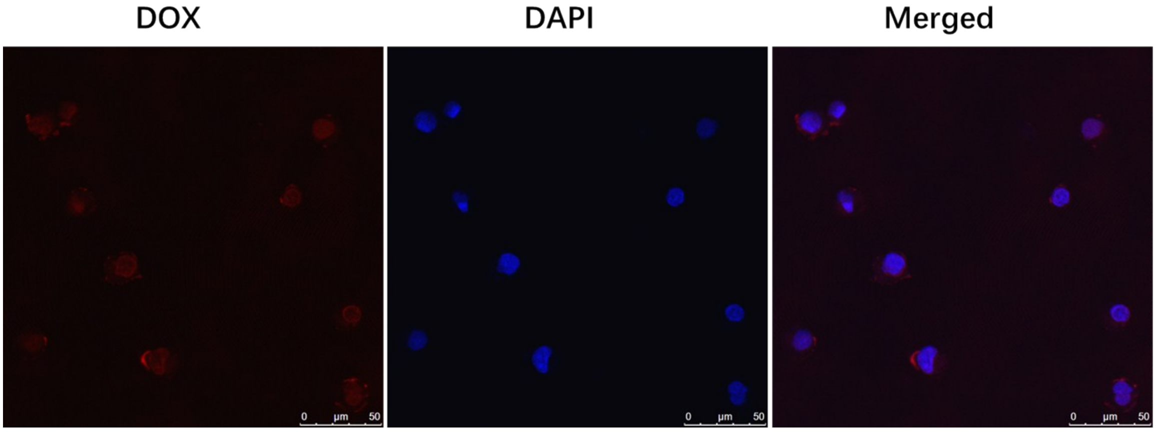

Fig. S6 CLSM images of K562/ADR cells incubated with the CAP/SiO_2/DOX@siRNA composite for 4 h. The red fluorescence from the DOX showed the location of the composite. The blue fluorescence was from cell nuclei stained with DAPI.

Real-time PCR assay for mRNA level of MDR1 gene

The forward and reverse primers targeting MDR1 sequence were 5'- GGAGGCCAACATACATGCCT-3', and 5'- AGGCTGTCTAACAAGGGCAC-3', respectively. The mRNA level of GAPDH gene was also measured in each sample as an internal normalization standard. The forward primer was 5'- CAAATTCCATGGCACCGTCA -3' and the reverse primer was 5′- AGCATCGCCCCACTTGATTT -3′. The PCR program was run on a Step One Plus Real-time PCR System (ABI, USA). All experiments were carried out in triplicate. Thermal cycling conditions were: 95 °C for 5min; 35 cycles of 95 °C/30 s, 58 °C/30 s, and 68 °C/30 s.

Western blot assay

Protein concentration was determined by BCA assay. 20 μg of total protein was boiled for 5 min in 4.5 μL of SDS sample buffer. The equal amount of proteins was loaded in each lane of 10% (w/v) SDS-polyacrylamide gel electrophoresis (SDS-PAGE) and then electrically transferred to a nitrocellulose membrane (NC membrane). After blocking the membranes with 5% (w/v) skimmed milk for 2 h, target proteins were immunoblotted with anti P –gp antibody and anti-GAPDH at 4 °C overnight. Appropriate horseradish peroxidase (HRP)-conjugated antibodies and samples were then detected by electrochemiluminescence.
